# Synthesis, characterization, molecular docking studies, and theoretical calculations of novel Ni (II), Cu (II), and Zn (II) complexes based on benzothiazole derivative

**DOI:** 10.1186/s13065-025-01576-1

**Published:** 2025-07-04

**Authors:** Mohamed G. Abd El-Nasser, Toka I. Ismail

**Affiliations:** Department of Chemistry, Faculty of Science, Helwan University, Cairo, 11795 Egypt

**Keywords:** Benzothiazole, DFT, TD-DFT calculations, Ni^2+^, Cu^2+^ and Zn^2+^ complexes, Molecular docking, Spectroscopic studies

## Abstract

Benzothiazole derivative (L) metal complexes with copper, nickel, and zinc have been synthesized. The structures of the prepared compounds were studied using various spectroscopic techniques, including ^1^H NMR spectroscopy, magnetic measurements, thermogravimetric analysis (TGA), electronic spectroscopy, and molar conductance. These techniques were employed to examine and validate the structures of the synthesized compounds. The copper and zinc complexes exhibit octahedral geometries, while the nickel complex adopts a distorted square planar geometry. The nonlinear optical (NLO) properties, geometrical optimization, and electronic transitions were theoretically investigated using the B3LYP time-dependent density functional theory (TD-DFT) with PCM (DMF as solvent). The LANL2DZ basis set was used for the metal ions, while the 6-311G** basis set was applied to carbon, hydrogen, and heteroatoms. The electronic configurations and natural charge populations were determined and reported using natural bond orbital (NBO) analysis. Global chemical reactivity descriptors were evaluated using the relatively small energy gap between the highest occupied Molecular orbital (HOMO) and the lowest unoccupied molecular orbital (LUMO), indicating the presence of intramolecular charge transfer (ICT) within the Zn^2+^, Cu^2+^, and Ni^2+^ complexes. The complexes exhibited intriguing optical properties, as suggested by their anisotropy of polarizability (α) and first-order hyperpolarizability (β) values. Furthermore, molecular docking studies revealed effective binding of the synthesized complexes to target proteins, including NI63 (Human Coronavirus), 1HK7 (Breast Cancer Protein), 6WTT (SARS-CoV-2), and 1ACL (Alzheimer’s Disease Protein).

**Graphical abstract:**

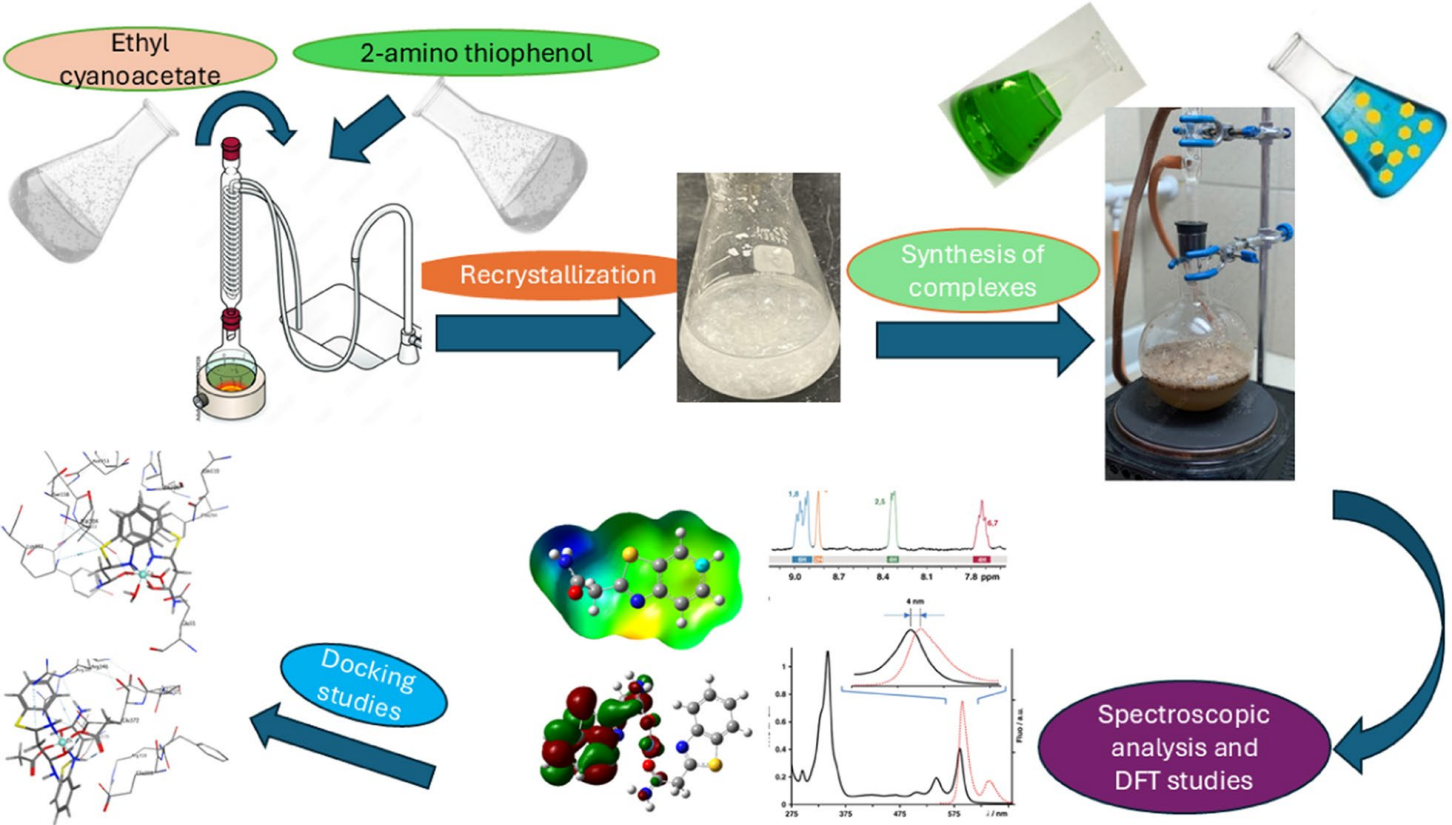

**Supplementary Information:**

The online version contains supplementary material available at 10.1186/s13065-025-01576-1.

## Introduction

Viruses that damage various human organs or tissues often result in a slow recovery. Due to genetic changes, viruses are becoming increasingly resistant [1, 2]. Additionally, bacterial infections that are resistant to multiple drugs pose a growing threat to public health. According to the UK government, antibiotic resistance causes the deaths of many thousands of people annually worldwide [3], and this number is projected to rise to 10 million by 2050 [4]. Bicyclic heterocyclic compounds, such as benzothiazole, are commonly found in bioactive molecules [5]. Benzothiazole has numerous modification sites that allow for the substitution of different heteroaryl groups, giving it a broad spectrum of biological activities, including antioxidant, anticancer, antibacterial, and anti-inflammatory effects [6–9].

Drug discovery is a cutting-edge field of medical science that is continuously seeking new ways to treat human diseases [10, 11]. Enzymes are attractive targets for pharmacological therapy due to their critical roles in biological processes involved in the pathophysiology of various diseases [12, 13]. Approximately 47% of all small-molecule drugs on the market target enzymes by inhibiting their activity [14]. Today, major pharmaceutical and biotechnology companies often focus their drug research and development efforts on enzymes because of their susceptibility to inhibition by small-molecule drugs [15]. Metal complexes typically bind to DNA through intercalation and electrostatic interactions in a non-covalent manner, often targeting the grooves of the DNA helix [16, 17]. To address the challenges in anticancer treatment, there is a pressing need for novel drugs that are non-covalently bonded, more affordable, and free from severe side effects. Owing to their diverse biological activities, the synthesis of a special class of metal complexes derived from azo dyes, hydrazones, and Schiff bases, especially those containing nitrogen (N) and sulfur (S) heteroatoms, has garnered significant interest [18–20].

The potential of various copper complexes acting as synthetic nucleases for cancer treatment has been extensively studied [21]. Several of these complexes have demonstrated remarkable efficacy, with biological activities superior to those of currently available anticancer drugs [22]. The cytotoxic and nuclease activities of the resulting metal complexes are known to be strongly influenced by the structure of the surrounding organic ligands [23]. The nature of the donor atoms and the structural characteristics of the complex can affect its lipophilicity or hydrophilicity, the preferred oxidation state of the metal center, and, most importantly, its biological activity [24, 25]. As an alternative class of chemotherapeutics, complexes of other transition metals are also being widely investigated [26, 27]. For example, two copper complexes and three ruthenium complexes have progressed to clinical trials [28]. Copper complexes are particularly attractive because endogenous metals like copper may be less harmful to healthy cells, potentially resulting in fewer toxic side effects [29]. Furthermore, because copper complexes may act through mechanisms distinct from those of traditional platinum-based drugs, they could help overcome the chemoresistance often associated with repeated platinum treatments [27].

This study aims to synthesize and characterize novel metal ion complexes based on a benzothiazole derivative. Additionally, time-dependent density functional theory (TD-DFT) calculations are used to investigate their computational parameters, and molecular docking is employed to predict binding conformations and free binding energies between the prepared complexes and selected target proteins.

## Experimental

### Materials, methods, and instrumentation

High-purity chemicals were used for all applications. These included nickel chloride hexahydrate, copper nitrate hydrate, ethanol, zinc acetate, ethyl acetoacetate, 2-aminothiophenol, and 30% ammonium hydroxide. FT-IR spectra were recorded using a Nicolet 380 spectrophotometer, covering the 450–4000 cm^−1^ range. Melting points were measured with an SMP3 melting point apparatus. The ^1^H NMR spectra were obtained using a Bruker Avance III 400 MHz spectrometer at Ain Shams University in Cairo, Egypt. Chemical extracts were analyzed, and the molecular weights of the isolated peaks were simultaneously determined using electrospray ionization (ESI) mass spectrometry combined with ultra-performance liquid chromatography (UPLC). Samples (100 μg/mL) were diluted in analytical-grade methanol and filtered through a 0.2 μm membrane disc filter before high-performance liquid chromatography (HPLC) analysis, which was conducted using a reversed-phase C18 column and 0.1% aqueous formic acid. Mass spectra were acquired in the ESI region, within the m/z range of 100–1000, using MassLynx 4.1 software. Provisional compound identification was performed by comparing the fragmentation patterns and retention times (Rt) of the mass spectra with previously reported data [30].

The synthesized metal complexes were subjected to thermal analysis using a Shimadzu TG–DTA analyzer (Japan) at a heating rate of 10 °C/min, with weight loss measured up to 1000 °C. The molar conductance of the complexes was determined in 10^–3^  M dimethylformamide (DMF). Magnetic properties were measured at room temperature using an MK1 Magnetic Susceptibility Balance. The electronic absorption spectra of the compounds were recorded in DMF using a Beckman spectrophotometer [31, 32].

### Synthesis of 2-(benzo[d]thiazol-2-yl)acetamide (L)

**Scheme 1.**
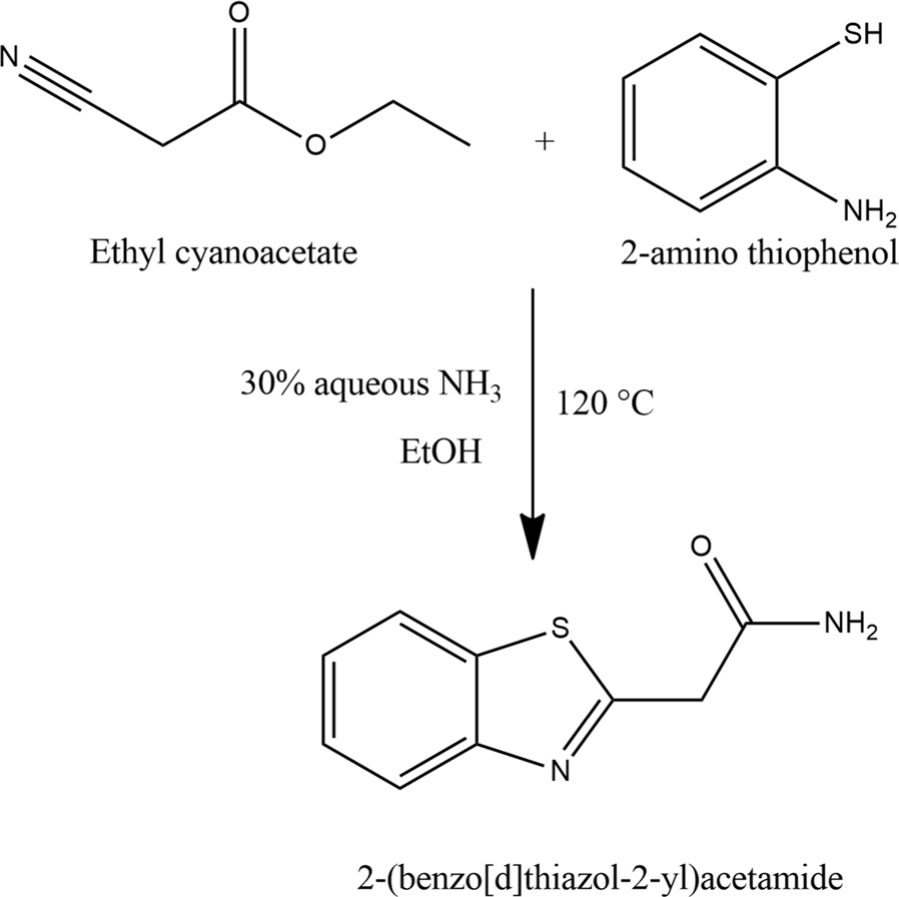
The reaction strategy for the prepared ligand (L)

The ligand was synthesized according to a previously reported method, as described below [33]. Ethyl cyanoacetate (6.00 g, 53.0 mmol) and 2-aminothiophenol (6.63 g, 53.0 mmol) were combined in an equimolar ratio and stirred for two hours at 120  °C under a nitrogen atmosphere. The reaction mixture was then allowed to cool to room temperature. After being obtained as a yellow oil (11.07 g, 50.0 mmol, 94%) and confirmed for purity by TLC, the product was subjected to further purification [34]. Approximately 11.60 g (52.4 mmol) of the compound was dissolved in 40 mL of 30% aqueous NH_3_ and 20 mL of ethanol. The mixture was stirred for 18 h at room temperature. The resulting yellow-green solid was collected as a pure precipitate (8.01 g, 41.7 mmol, 80%) with a melting point of 168 °C. The reaction strategy for the prepared ligand is illustrated in Scheme 1.

### Synthesis of metal complexes (ML_2_)

The complexes were prepared by mixing an ethanolic solution of 0.01 mol of the metal ion with 0.02 mol of the ligand. The mixtures were refluxed for approximately six hours with continuous stirring, then allowed to dry. The resulting colorful 1:2 metal-to-ligand complexes were purified by recrystallization from ethanol. After filtration under suction, the solids were repeatedly washed with appropriate solvents until the filtrate was clear. The metal ion content in the resulting solid complexes was determined by titration with EDTA using suitable indicators. A summary of the analytical and physicochemical studies performed to confirm the structures of the synthesized complexes is provided in Table 1.

**Table 1:** Molar conductance (Λ_m_), effective magnetic moment (μ_eff_), and elemental analysis of the ligand (L) and its Zn^2+^, Cu^2+^, and Ni^2+^ complexes

L/ML_2_	Empirical formula	M. wt	C% (Calc.) found	H% (Calc.) found	N% (Calc.) found	S% (Calc.) found	M% (Calc.) found	μ_eff_	Λ_m_
L	C_9_H_8_N_2_OS	192.24	(56.23)	(4.19)	(14.57)	(16.68)	–	–	-
			56.51	3.96	15.02	16.87	–	–	-
[Ni(L)_2_]2Cl.4H_2_O	C_18_H_24_Cl_2_N_4_O_6_S_2_Ni	586.14	(36.88)	(4.13)	(9.56)	(10.94)	(10.01)	0.00	190
			36.61	4.48	9.32	11.08	10.33		
[Cu(L)_2_.(OH_2_)_2_]2NO_3_.2H_2_O	C_18_H_24_N_6_O_12_S_2_Cu	644.09	(33.57)	(3.76)	(13.05)	(9.96)	(9.87)	2.05	172
			32.98	4.03	13.22	10.26	9.49		
[Zn(L)_2_.(OAc)_2_]3H_2_O	C_22_H_28_N_4_O_9_S_2_Zn	621.99	(42.48)	(4.54)	(9.01)	(10.31)	(10.51)	0.00	10.60
			42.79	4.89	9.11	10.46	10.39		

### Computational models

The structures of the ligand and its complexes were determined through energy minimization calculations using the Gaussian 09W software package [35]. The geometrical structures of the ground states were optimized using the DFT method with the B3LYP exchange–correlation functional. The 6-311G** basis set was applied for C, N, S, Cl, O, and H atoms, while the LANL2DZ basis set was used for the metal ions. All structures were fully optimized in the gas phase without applying any symmetry constraints [36].

The full capabilities of the GaussView 5 program were utilized to generate molecular orbital (MO) figures [37]. The three-dimensional coordinates, along with the B3LYP theoretical approach, were used to calculate the nonlinear optical (NLO) properties ∆α, µ, < β > , and < α > . The obtained FT-IR spectra were compared with theoretical calculations of the harmonic vibrations for the synthesized ligand and the prepared complexes. Additionally, the electronic spectra of the generated molecules were calculated and simulated using TD-DFT [38–40].

### Molecular docking

Using the docking program MOE 2019, the interactions and probable binding sites between small molecules (ligands or complexes) and nucleic acids (DNA) were investigated. The Protein Data Bank (PDB) was used to obtain X-ray crystal structures for several target proteins, including the Alzheimer's disease protein (PDB ID: 1ACL), SARS-CoV-2 (6WTT), breast cancer protein (1HK7), and human coronavirus NL63 (5EPW). The binding interactions between these proteins and the synthesized complexes were also explored.

Docking simulations were performed using default settings. Conformations were selected based on the E conformation, S score values, and optimal fitting with key amino acid residues in the binding pocket. Interestingly, the active site geometries and conformations of these proteins closely resemble those of the studied ligand and its complexes, making them strong candidates for accurate binding predictions [41].

## Results and discussion

### FT-IR spectra of the Prepared Ligand and its complexes

In the FT-IR spectrum of the ligand, the weak band observed around 3375 cm^−1^ is attributed to NH_2_ stretching vibrations, while the strong band at 1667 cm^−1^ corresponds to the C = O group of the free ligand. A medium-intensity band at 1623 cm^−1^ is assigned to the imine (C = N) group. Additionally, the band appearing at 3192 cm^−1^ in the infrared spectra of the compound is due to the aromatic C–H stretching vibration [42]. Table 2 summarizes both the ligand's calculated and experimental FT-IR bands and its complexes.

**Table 2:** Theoretical and experimental FT-IR bands of the ligand and its complexes with Ni^2+^, Cu^2+^, and Zn^2+^

Band Assignment						
Ligand/Complex	**ν**_**OH**_ Exp. (Theo.)	**ν**_**NH2**_ Exp. (Theo.)	**ν**_**C=N**_ Exp. (Theo.)	**ν**_**C=O**_ Exp. (Theo.)	**ν**_**M–N**_ Exp. (Theo.)	**ν**_**M-O**_ Exp. (Theo.)
L	–	3375 (3617)	1623 (1627)	1667 (1865)	–	–
Ni-L (1:2)	3258 (–)	3421 (3589)	1603 (1629)	1651 (1735)	481 (491)	538 (537)
Cu-L (1:2)	3189 (3154)	3430 (3584)	1610 (1639)	1669 (1733)	476 (483)	585 (570)
Zn-L (1:2)	3389 (3273)	3490 (3589)	1543 (1629)	1625 (1735)	460 (415)	530 (504)

**Fig. 1:**
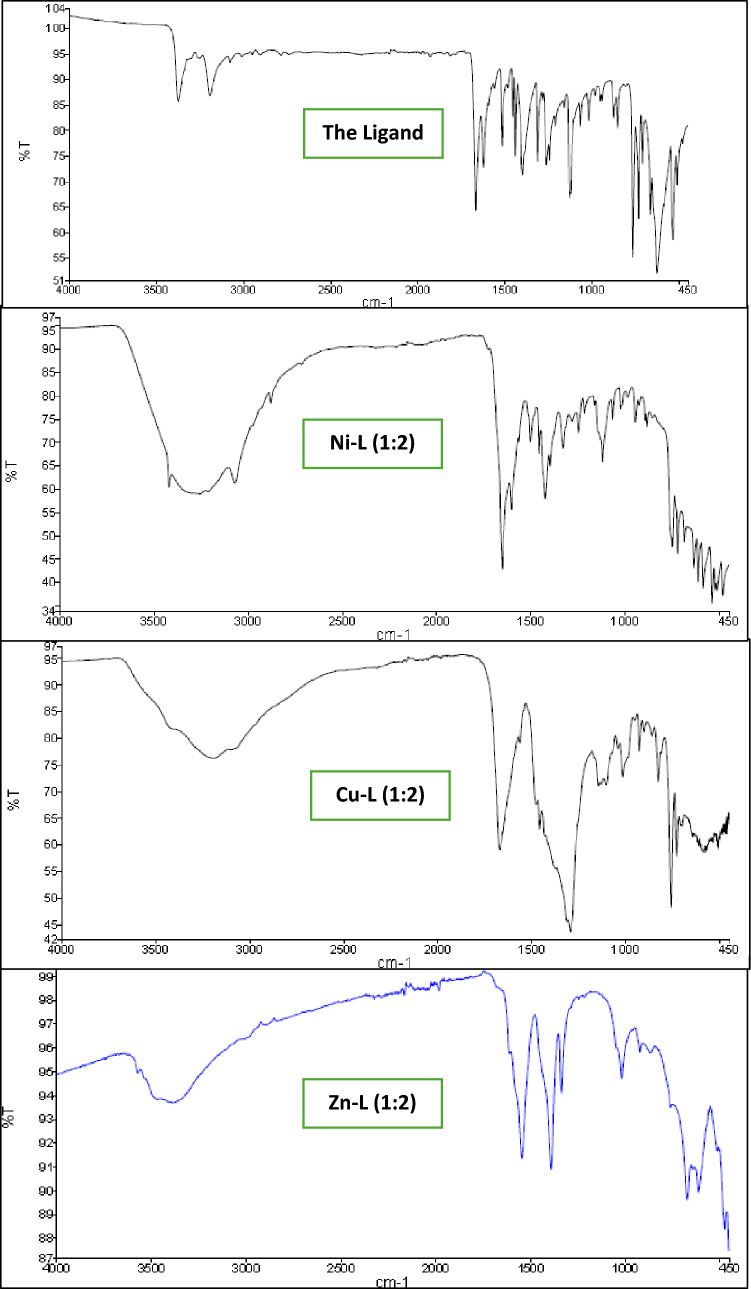
FT-IR spectra of the prepared Ligand and its complexes

For the Ni, Cu, and Zn complexes, the broad band observed in the 3189–3389 cm^−1^ range is attributed to the O–H stretching vibration of both coordinated and hydrated water molecules. Chelation with the metal ions causes the C = N stretching band, typically observed around 1600–1610 cm^−1^, and the C = O stretching band at 1651–1669 cm^−1^, to broaden and shift. The bands appearing in the ranges of 459–481 cm^−1^ and 530–585 cm^−1^ in the infrared spectra of the chelates are assigned to the stretching vibrations of nitrogen and oxygen atoms coordinated to the metal ions, respectively. Additionally, the band observed at 1388 cm^−1^ is attributed to the stretching vibration of the acetate group coordinated to the zinc ion [43, 44]. The FT-IR spectra of the synthesized ligand and its complexes are shown in Fig. 1.

**Scheme 2.**
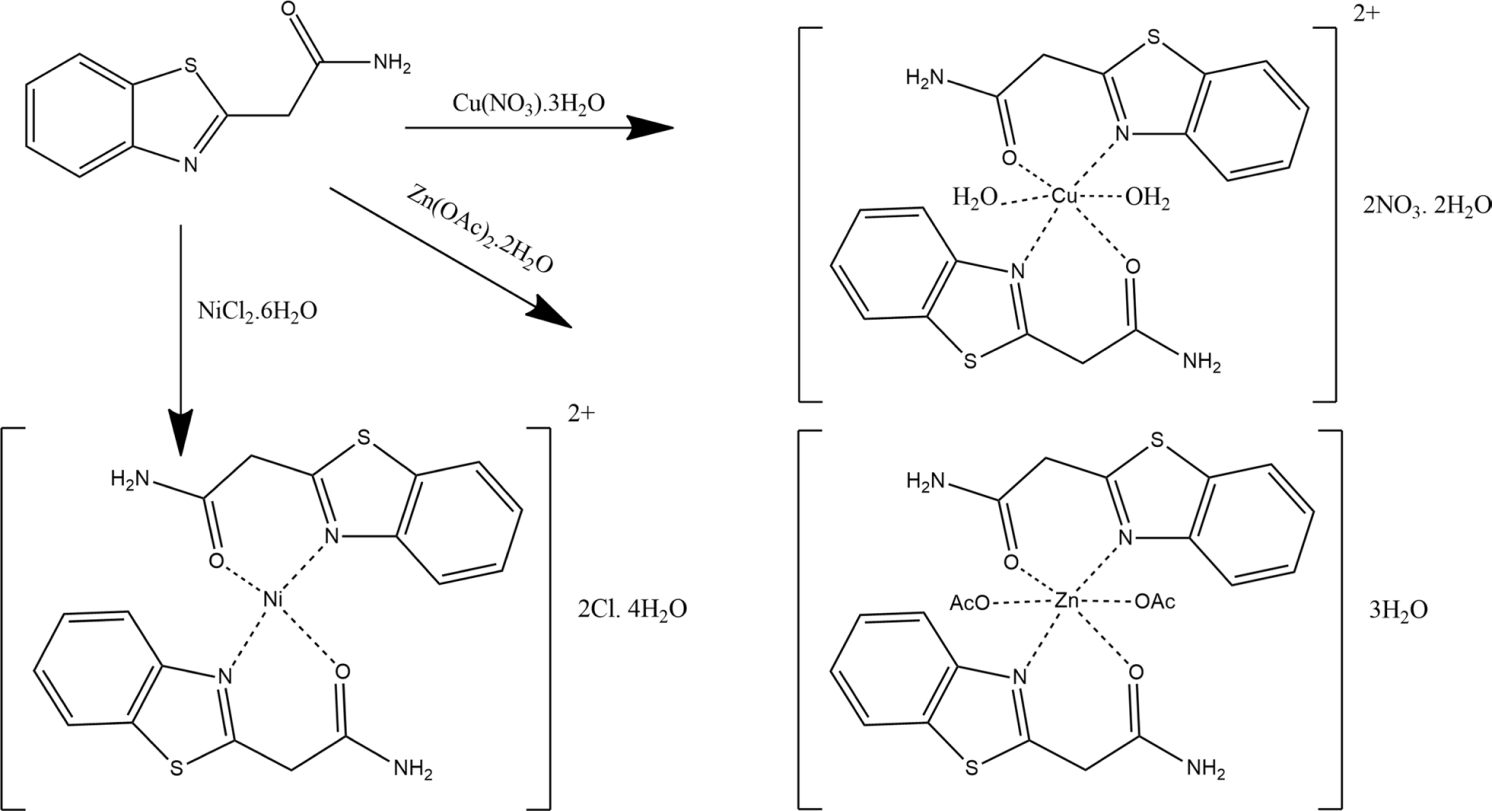
Reaction strategy and structural formulas of the investigated Ni(II), Cu(II), and Zn(II) complexes

Scheme 2 provides a summary of the structures of the synthesized ligand and its complexes based on spectroscopic investigations, magnetic moment measurements, and elemental analysis. The proposed stereochemical configurations of the metal chelates under investigation suggest square planar geometry for Ni-L and octahedral geometry for Cu-L and Zn-L.

### ^1^H NMR spectra of the ligand and its complex with zinc

The molecular structures of the ligand and Zn–L complex were analyzed using ^1^H NMR chemical shifts. The ^1^H NMR spectrum of the ligand shows sharp peaks, including a doublet of doublets (J = 8 Hz) and triplets (J = 8 Hz), between 7.46 and 8.06 ppm, corresponding to aromatic protons. A broad signal appears at 7.24 ppm, attributed to the proton attached to the amide group. A sharp singlet observed at 4.02 ppm is assigned to the methylene protons.

The ^1^H NMR spectrum of the Zn–L complex shows a slight shift of the methylene protons from 4.02 ppm in the free ligand to 4.15 ppm in the complex. A new singlet signal at 1.81 ppm appears, corresponding to the acetate ions coordinated to the metal ion. Additionally, a broad singlet at 3.35 ppm is attributed to the protons of coordinated water molecules in the complex [45]. Due to the absence of protons in the donor groups at the coordination center, the effect of coordination with Zn^2+^ can be observed through the slight shift and broadening of the signals of the groups located near the coordination center.

**Fig. 2.**
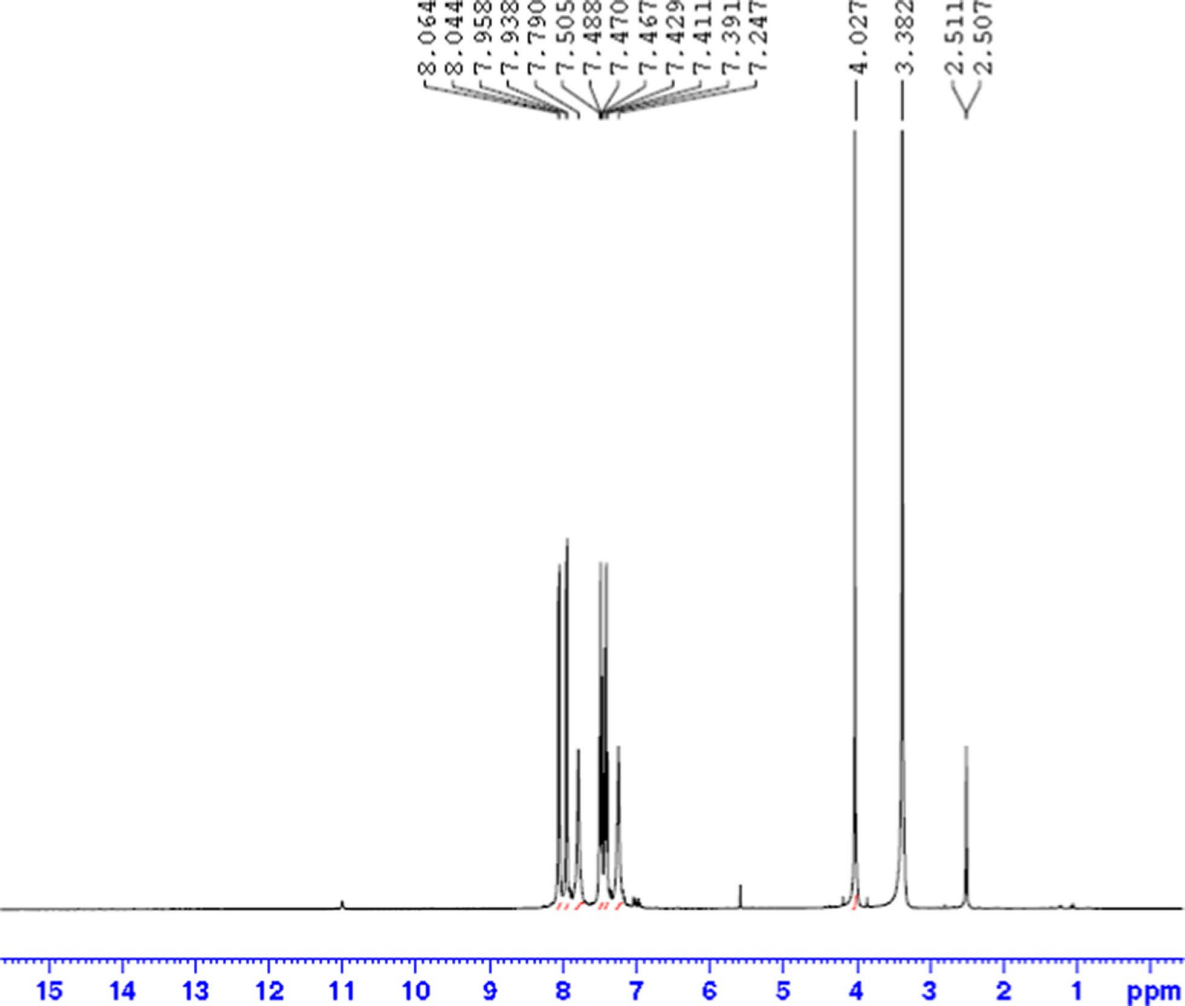
^1^H NMR spectrum for the prepared ligand (L)

**Fig. 3.**
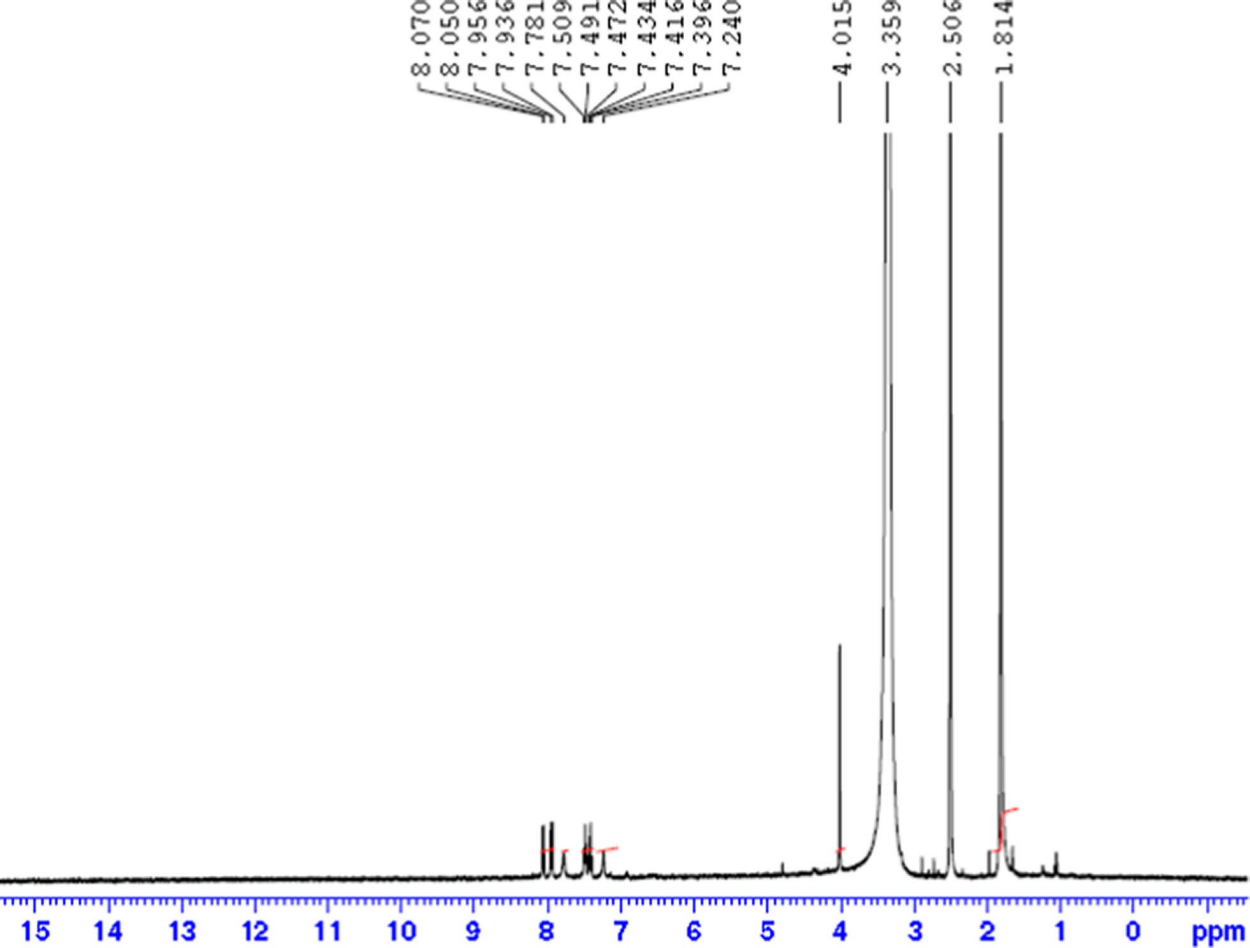
^1^H NMR spectrum for the Zn-L (1:2) Complex

Figures 2 and 3 show the ^1^H NMR spectra of the ligand and Zn-L complex, respectively, while Table 3 summarizes their spectral data.

**Table 3:** ^1^H NMR spectral data for the ligand and its zinc complex

Complex (M:L)	Chemical shift(δ)	Assignment
L	7.46–8.06	Aromatic C-H protons
	7.24	NH_2_
	4.02	CH_2_
Zn-L (1:2)	7.39–8.07	Aromatic C-H protons
	7.24	NH_2_
	4.15	CH_2_
	3.35	H_2_O coordinated with Zn
	1.81	CH_3_ of the acetate group

### Studies of the ligand's mass spectra L and its complexes

The ESI (+ ve) spectrum of the ligand L (C_9_H_8_N_2_OS) shows a peak at m/z = 193.26, corresponding to [L + H]^+^ (Calculated m/z = 192.24). Another peak at m/z = 215.01 is attributed to [L + Na]^+^. The analysis of the ESI spectrum, along with data obtained from FT-IR and ^1^H NMR, confirms the proposed structure of the ligand as shown in Scheme 1.

The mass spectrum of the Ni complex shows a peak at m/z = 551.18, corresponding to [2L + 4H_2_O + NiCl]^+^ (C_18_H_24_ClN_4_NiO_6_S_2_; calculated m/z = 550.68). A peak at m/z = 421.02 corresponds to [L + 4H_2_O + 2MeOH + NiCl]^+^ (C_11_H_24_ClN_2_NiO_7_S; calculated m/z = 422.53). The mass spectrum of the Cu complex shows a peak at m/z = 439.98, assigned to [L + 5H_2_O + MeOH + Cu(NO_3_)]^+^ (C_10_H_22_CuN_3_O_10_S; calculated m/z = 439.91). Another peak at m/z = 953.95 is observed due to [3L + 2MeOH + 2Cu^2+^  + 3NO_3_^−^] (C_29_H_32_Cu_2_N_9_O_14_S_3_; calculated m/z = 953.90).

**Fig. 4:**
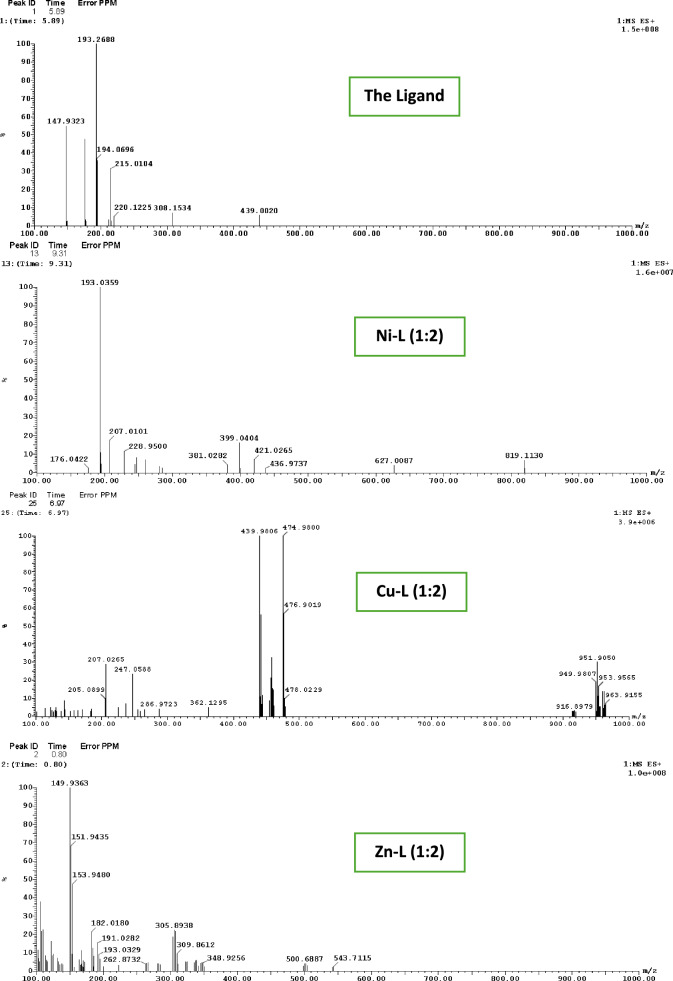
ESI mass spectra of the investigated ligand and its complexes

The mass spectrum of the Zn complex shows a peak at m/z = 348.92, attributed to [L + MeOH + Zn^2+^  + (OAc)^−^] (C_12_H_15_N_2_O_4_SZn; calculated m/z = 348.70). Additional peaks are observed at m/z = 543.71 for [2L + 2H_2_O + Zn^2+^  + (OAc)^−^] (C_20_H_23_N_4_O_6_S_2_Zn; calculated m/z = 544.93), and at m/z = 595.11 for [2L + 3H_2_O + MeOH + Zn^2+^  + (OAc)^−^] (C_21_H_29_N_4_O_8_S_2_Zn; calculated m/z = 594.99) [30, 46, 47]. Figure 4 shows the selected ESI spectra of the ligand and its complexes, while Figure S1 presents the detailed mass spectra of the prepared compounds.

### Thermogravimetric analysis (DTG and TGA) of the ligand and its complexes

Thermal studies of the Ni-L complex were conducted in real-time using TGA-DTA analysis. Table 4 summarizes the calculated and experimental mass losses. Between 90 and 150 °C, the Ni complex exhibits a weight loss of 12.38% (calculated: 12.29%), corresponding to the loss of four water molecules. A subsequent weight loss of 11.90% (calculated: 12.09%) is observed between 290 and 400  °C, which is attributed to the elimination of two chloride ions.

**Table 4:** TGA/DTA experimental data for Ni^2+^, Zn^+2^, and Cu^2+^ complexes with the ligand L

Complexes	M. Wt	Temp (°C)	Calc Loss %	Found Loss %	Assignment
Ni-L (1:2)	586.14	90–150	12.29	12.38	Four hydrated water molecules are lost
		290–400	12.11	11.90	Removal of two chlorine ions
		460–670	65.59	61.46	Organic ligand loss
		> 670	12.74	14.28	NiO residue that remains
Cu-L (1:2)	644.09	70–150	5.59	6.04	Two hydrated water molecules are lost
		160–250	24.22	24.8	Loss of (HNO_3_ + O_2_)
		550–950	12.35	13.33	Remaining CuO residue
Zn-L (1:2)	621.99	85.5–150	8.69	7.75	Three hydrated water molecules are lost
		265–344	18.98	18.87	Two coordinated acetate groups are lost
		346–415	61.81	60.17	Destruction of the organic molecule
		> 415	13.15	14.27	Remaining ZnO residue

A significant weight loss occurs between 460 and 670 °C, as indicated by the DTA thermogram, corresponding to the complete decomposition of the organic ligand. Above 670  °C, the decomposition proceeds slowly, yielding a final residue of 14.28% (calculated: 12.74%) between 670 and 800  °C, which corresponds to the formation of nickel oxide. This thermal behavior supports the proposed stoichiometry of the complex [48–50].

Between 85.5 and 150 °C, the Zn-L complex loses 7.75% of its mass (calculated: 8.69%), corresponding to the loss of three water molecules. Two coordinated acetate groups are removed between 265 and 344  °C, resulting in a weight loss of 18.87% (calculated: 18.98%). A significant weight loss is observed above 346  °C, as confirmed by the DTA thermograms, corresponding to the decomposition of the organic moiety. Above 415  °C, the decomposition proceeds gradually due to the formation of zinc oxide, leaving a residual mass of 14.27% (calculated: 13.15%).

For the Cu-L complex, two coordinated water molecules are lost between 70 and 150 °C, resulting in a mass loss of 6.04% (calculated: 5.59%). A further weight loss of 24.8% (calculated: 24.22%) occurs between 160 and 250 °C, corresponding to removing HNO_3_ and O_2_. Above 550 °C, gradual decomposition occurs due to the formation of copper oxide, resulting in a final residue between 550 and 950 °C of 13.33% (calculated: 12.35%) [48, 51].

**Fig. 5:**
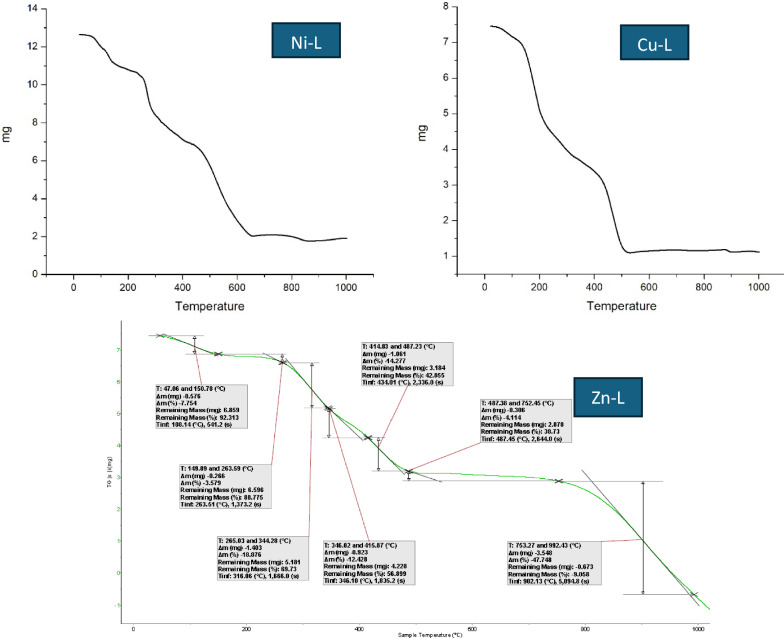
TGA spectra of the prepared complexes

Figure 5 shows the TGA spectra of Ni-L, Cu-L, and Zn-L complexes.

### Molar conductivity measurements

In 10^–3^ M DMF, the Zn-L complex shows a molar conductivity of 10.60 Ω^−1^ cm^2^ mol^−1^, indicating that the acetate groups are coordinated to the metal ion rather than as free ionic species. In contrast, the Ni-L and Cu-L complexes exhibit molar conductivity values ranging from 172 to 190 Ω^−1^ cm^2^ mol^−1^ [52]. When the Ni-L complex was treated with silver nitrate, a white precipitate formed, confirming the presence of chloride as a counterion [53]. Table 1 summarizes the experimental molar conductivity values for each of the complexes under investigation.

### Electronic absorption, magnetic susceptibility, and molar conductance

The magnetic moments of the complexes were calculated from the magnetic susceptibility measured using a Gouy magnetic balance, based on the apparent change in weight of the complexes when placed in a magnetic field. The effective magnetic moment was calculated using the equation μ_eff_ = 2.828 (X_m_ T)^1/2^B.M., where χ_m_ is the molar magnetic susceptibility corrected for the diamagnetism of all atoms in the compounds using Selwood’s and Pascal’s constants [54, 55].

The prepared ligand’s electronic absorption spectra show broad bands around 320–350 nm, attributed to π-π* transition within the C_6_H_5_ ring, and around 380–390 nm, due to n-π* transitions associated with the C = O group. The presence of a very weak peak in the Ni-L spectrum at 450 nm suggests that the strong metal-to-ligand charge transfer (MLCT) transition outweighs the contribution of the d-d transition characteristic of the complex’s square planar structure [36, 56].

A band was observed at 340 nm due to LMCT, and the Ni complex exhibited diamagnetic properties [45]. Based on the electronic absorption spectrum of the divalent Cu metal ion, an octahedral geometry was proposed around the central metal ion, showing ^2^A_2g_ → ^2^T_1g_ transitions at 220–250 nm, 401 nm, and a broad band at 430 nm [41, 57]. For the Zn(II) complex, the broad, intense band observed around 350 nm in the electronic spectrum was attributed to ligand-to-metal charge transfer. The complex exhibits diamagnetic characteristics, as expected, and adopts an octahedral geometry [32]. The zinc complex is non-electrolytic, as supported by its low molar conductance value shown in Table 1.

### Molecular orbital calculations

**Fig. 6:**
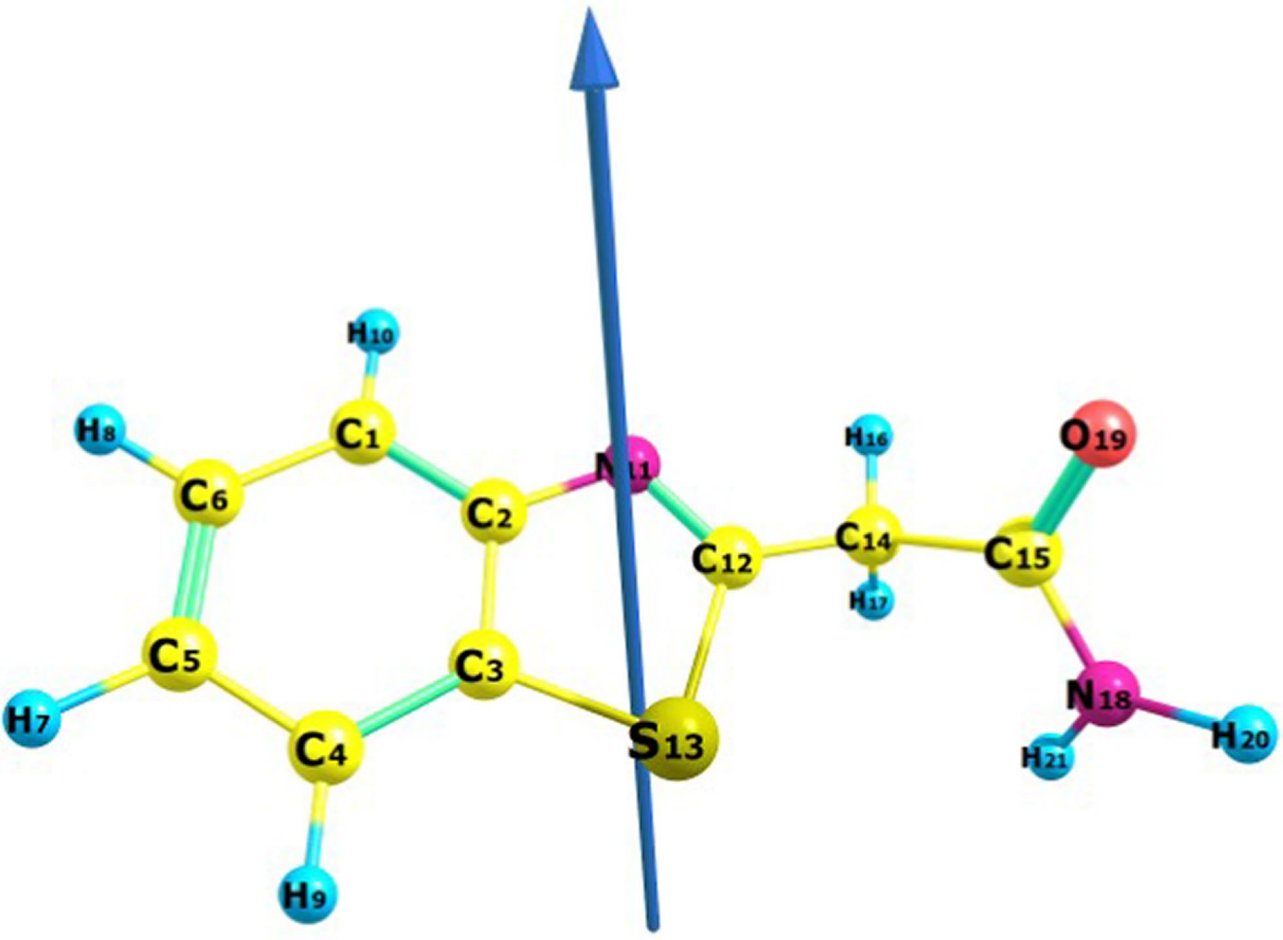
Bond lengths, dipole moment vector, numbering system, and optimized geometry of the ligand under study, calculated at the B3LYP/6-311G** level

**Fig. 7:**
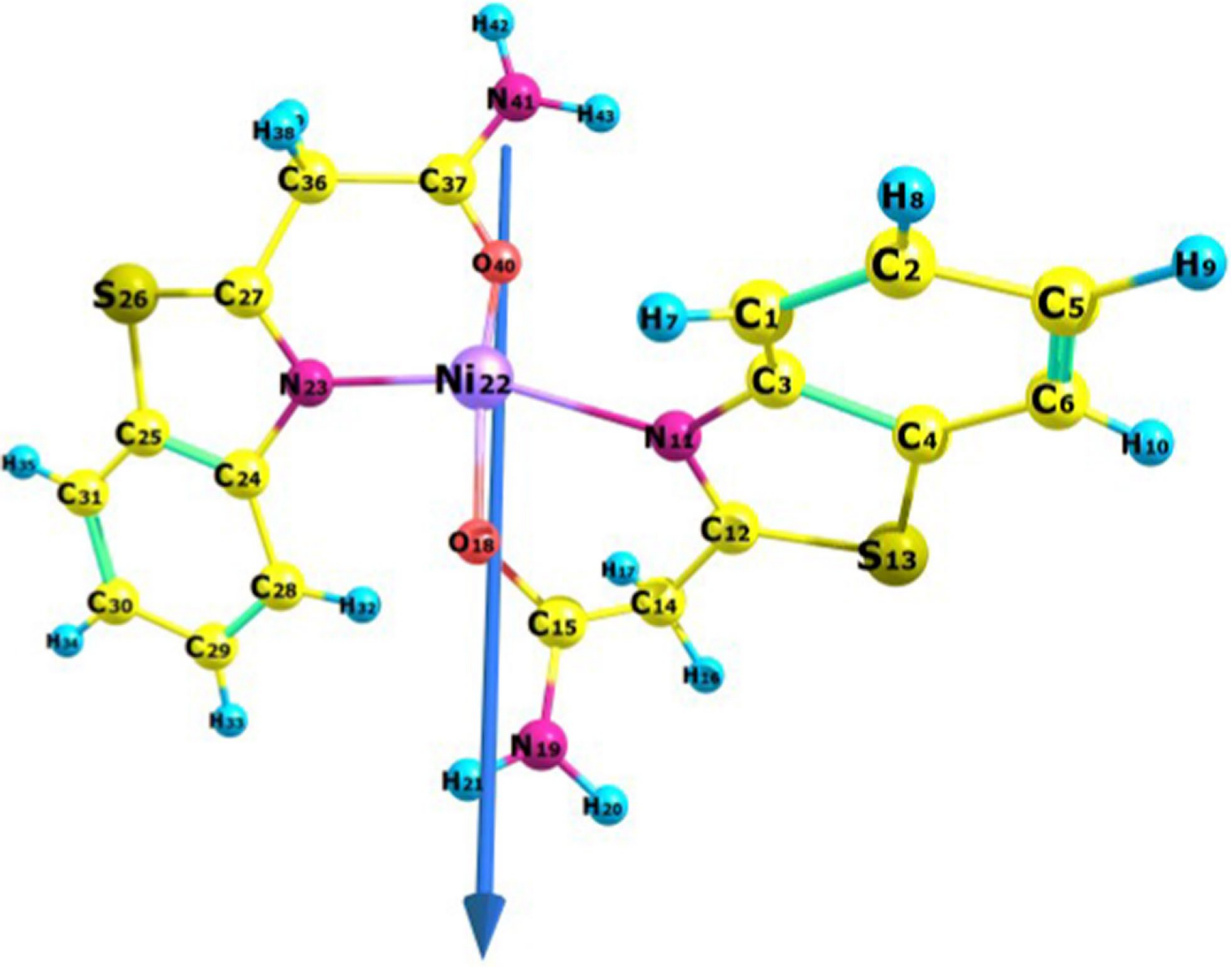
Bond lengths, dipole moment vector, numbering system, and optimized geometry of the Ni complex under study, calculated at the B3LYP/6-311G** level

**Fig. 8:**
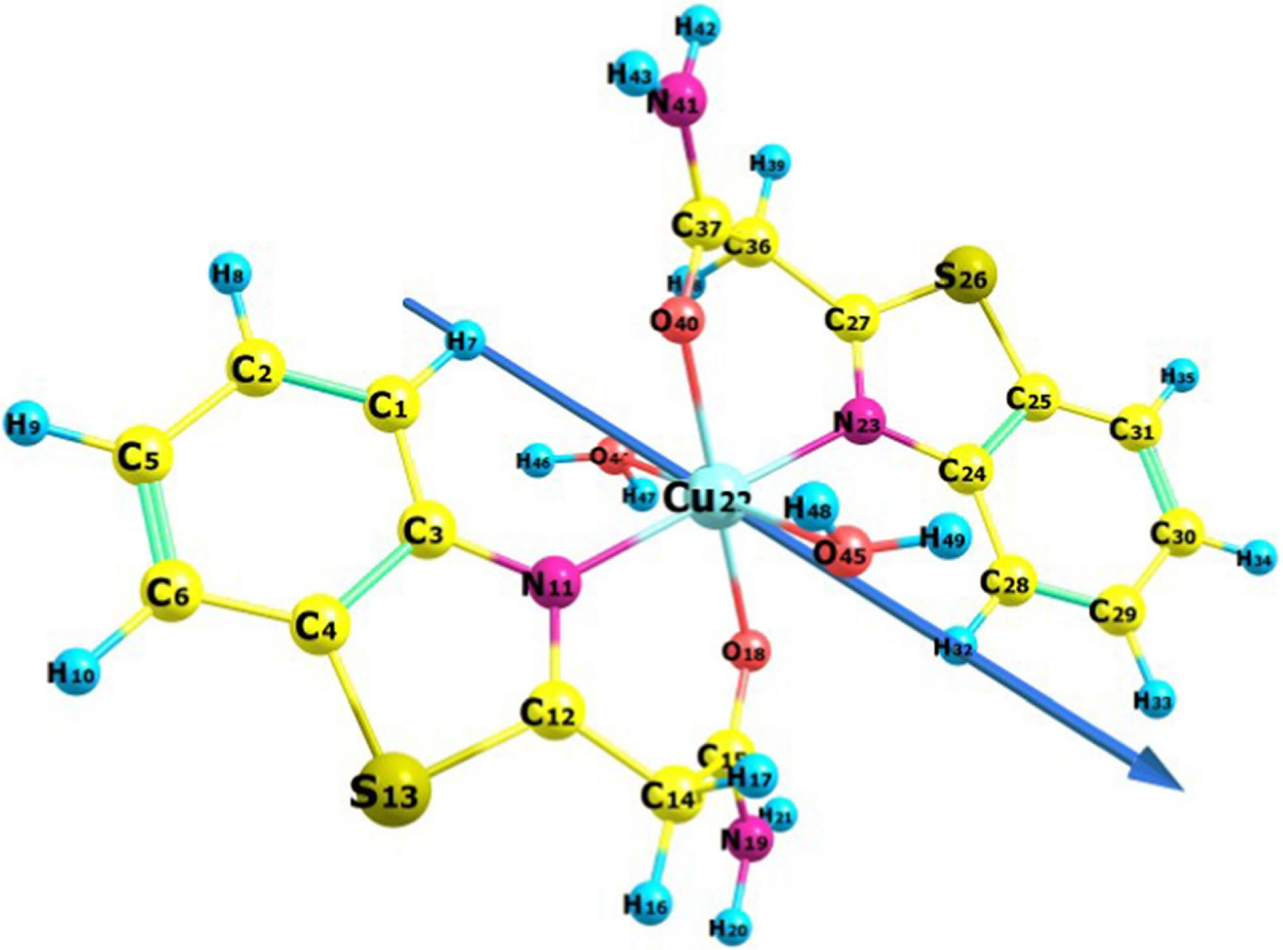
Bond lengths, dipole moment vector, numbering system, and optimized geometry of the Cu complex under study, calculated at the B3LYP/6-311G** level

**Fig. 9:**
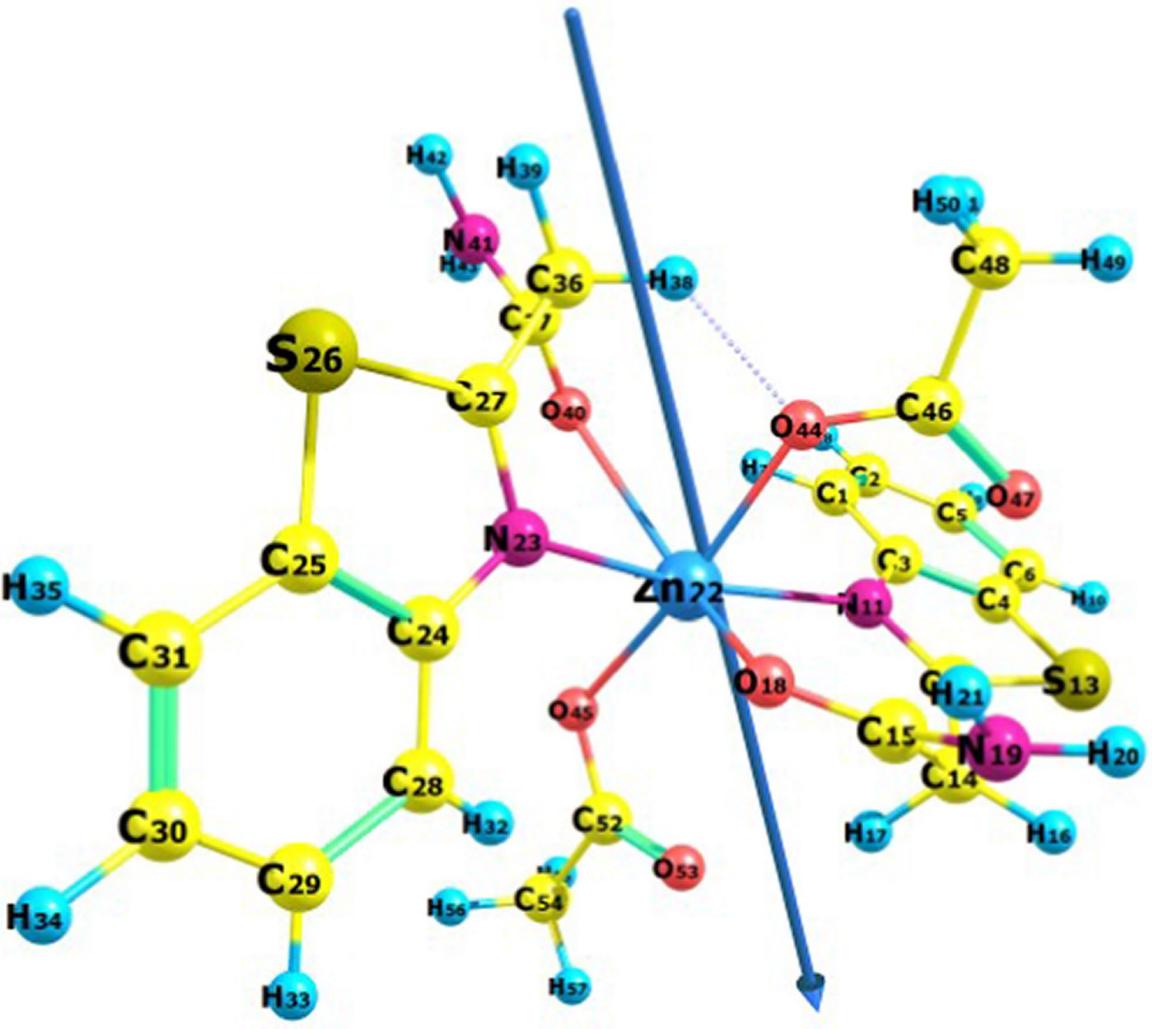
Bond lengths, dipole moment vector, numbering system, and optimized geometry of the Zn complex under study, calculated at the B3LYP/6-311G** level

Optimal investigations of the geometric properties of the materials under study were conducted using B3LYP-level theory for the Ni, Cu, and Zn complexes. The geometrical properties (bond angles, bond lengths, and dihedral angles), NBO analysis (natural populations, natural charges, and natural configurations), and electrostatic potential were computed. The optimized structure and numbering scheme of the ligand are shown in Fig. 6. Figures 7, 8,9 present the optimized structures and numbering schemes of the complexes studied. In the case of Cu-L, an octahedral structure is formed by coordinating the copper ion with two ligand molecules and two water molecules. In contrast, for Zn-L, the octahedral geometry consists of two ligand molecules and two acetate ions, according to theoretical findings. Zinc, copper, and nickel ions are coordinated to the ligands via N_11_, O_18_, N_23_, and O_40_ [51, 58–61].

Table 5 shows the calculated parameters of the prepared compounds.

**Table 5:** Bond angles, bond lengths, and dihedral angles of the prepared ligand and complexes

		Bond length		Bond angle		Dihedral angle	
	L	C_15_–N_18_	1.36620	N_18_–C_15_–O_19_	123.048	H_20_–N_18_–C_15_–O_19_	1.199
		C_15_–O_19_	1.21153	N_18_–C_15_–C_14_	114.835	N_18_–C_15_–C_14_-C_12_	102.057
		C_12_–N_11_	1.28704	S_1_–C_12_–N_11_	115.389	C_3_–S_13_–C_12_–N_11_	0.101
		C_12–_S_13_	1.78539	C_2_–N_11_–C_12_	111.708	C_2_–N_11_–C_12_–S_13_	0.008
				C_3_–S_13_–C_12_	88.465		
	Ni–L (1:2)	O_18_–Ni	1.85157	O_18_–Ni–N_23_	90.508	O_18_–Ni–N_23_–C_27_	20.844
		O_40_–Ni	1.35682	O_18_–Ni–O_40_	170.417	O_18_–Ni–O_40_–C_37_	−136.977
		N_11_– Ni	2.26604	O_18_–Ni–N_11_	72.292	O_18_–Ni–N_11_–C_3_	− 121.342
		N_23_–Ni	2.03305	N_23_–Ni–N_11_	162.079	N_23_–N–N_11_–C_27_	160.183
				N_23_–Ni–O_40_	98.377		
	Cu–L (1:2)	O_18_–Cu	2.04194	O_18_–Cu–N_23_	92.884	O_18_–Cu–N_23_–C_27_	143.639
		O_40_–Cu	2.04268	O_18_–Cu–O_40_	178.752	O_18_–Cu–O_40_–C_37_	− 22.533
		N_11_– Cu	2.08096	O_18_–Cu–N_11_	86.668	O_18_–Cu–N_11_–C_3_	− 142.761
		N_23_–Cu	2.07320	N_23_–Cu–N_11_	179.252	N_23_–Cu–N_11_– C_12_	− 18.636
		O_44_–Cu	2.43634	N_23_–Cu–O_40_	86.445	N_23_–Cu–O_40_–C_37_	34.986
		O_45_–Cu	2.42634	O_44_–Cu–N_23_	90.282	O_44_–Cu–N_23_–C_24_	− 125.788
				O_45_–Cu–N_23_	91.179	O_45_–Cu–N_23_–C_24_	− 121.980
	Zn–L (1:2)	O_18_–Zn	2.15418	O_18_–Zn–N_23_	77.931	O_18_–Zn–N_23_–C_27_	115.828
		O_40_–Zn	2.57906	O_18_–Zn–O_40_	145.810	O_18_–Zn–O_40_–C_37_	7.879
		N_11_– Zn	2.36156	O_18_–Zn–N_11_	95.901	O_18_–Zn–N_11_–C_3_	− 166.386
		N_23_–Zn	2.16994	N_23_–Z–N_11_	167.275	N_23_–Zn–N_11_– C_12_	75.922
		O_44_–Zn	2.12819	N_23_–Zn–O_40_	74.127	N_23_–Zn–O_40_–C_37_	44.230
		O_45_–Zn	2.22261	O_44_–Zn–N_23_	88.129	O_44_–Zn–N_23_–C_24_	− 136.830
				O_45_–Zn–N_23_	93.863	O_45_–Zn–N_23_–C_24_	53.272

### Natural charges and natural population

Table 6 displays the charges accumulated on each atom in the ligand’s natural electronic state. N_11_, N_23_, O_18_, and O_40_ carry the highest electronegative charges in the ligand under study. Electrons are typically attracted to the most electropositive cations. Figure S2 shows the cumulative charges of the compounds under investigation.

**Table 6:** Natural charges of the ligand (L) under investigation, calculated at the B3LYP/6-311G** level

Numbering system	L
N11	− 0.489
S13	0.209
N18	− 0.593
O19	− 0.527

The metal ions accept 1.242e (3d^8.66^), 0.993e (3d^9.32^), and 0.61e (3d^9.99^), for Ni, Cu, and Zn complexes, respectively, from the ligand’s donor atoms.

### Global reactivity descriptors

**Fig. 10:**
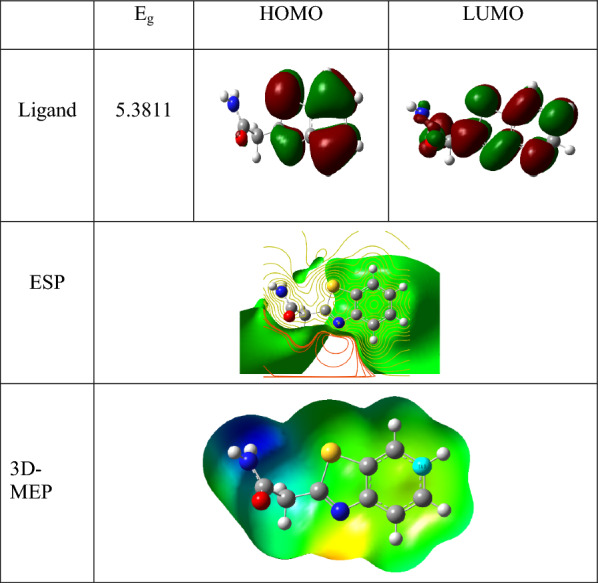
Frontier molecular orbitals, electrostatic potential, and 3D-MEP of the investigated ligand

**Fig. 11:**
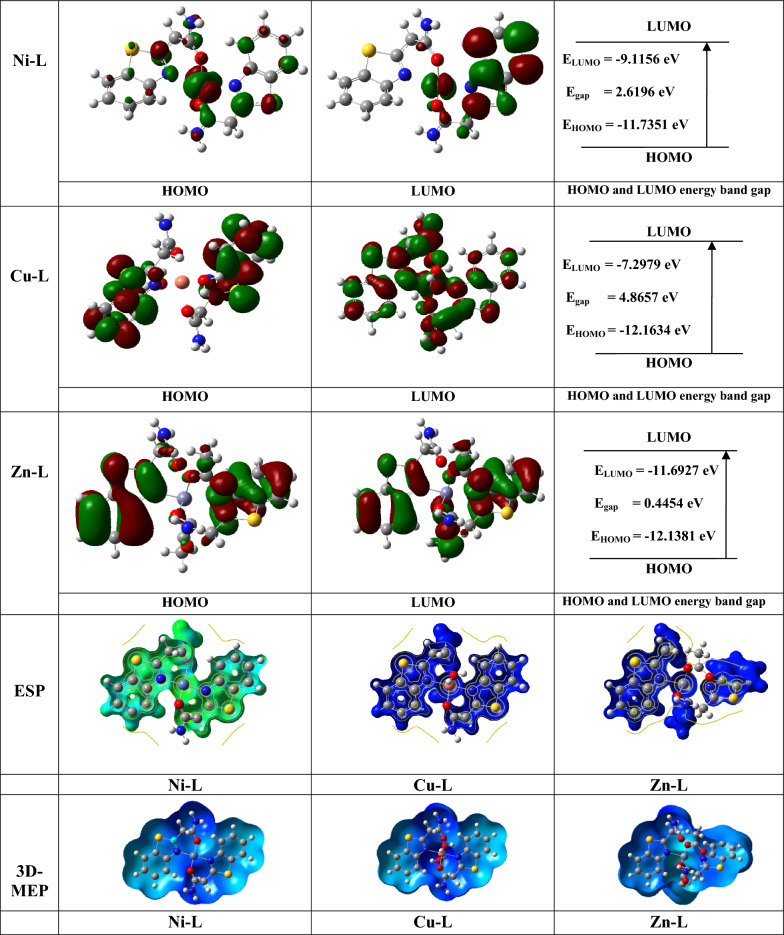
Frontier molecular orbitals, electrostatic potential, and 3D-MEP of the studied Zn, Cu, and Ni complexes

Tables 7 and 8, along with Figs. 10 and 11, display the frontier molecular orbital (FMO) energies of the synthesized ligand, optimized and calculated at the B3LYP/6.311G** level, and its complexes, calculated using B3LYP/LANL2DZ. The parameters include chemical potential (V), electronegativity (χ), chemical hardness (η), ionization potential (V), highest occupied molecular orbitals energy (E_HOMO_), lowest unoccupied molecular orbitals energy (E_LUMO_), energy gap (Eg), electron affinity (A), and chemical softness (S), all calculated using standard equations [62]. The chemical reactivity of molecules is defined by the (Eg). The data show that a lower energy gap facilitates charge transfer and polarization within the ligand [36, 38, 43, 62–64]. Tables 7 and 8 summarize the calculated parameters.

**Table 7:** Calculated quantum chemical parameters of the ligand

Compounds	Ligand
E_T_ (au)	− 930.8785
E_HOMO_ (eV)	− 6.6292
E_LUMO_ (eV)	− 1.2481
Energy band gap, E_gap_,eV, (E_HOMO_–E_LUMO_)	5.3811
µ (Debye)	4.0511
I, eV, (-E_HOMO_)	6.6295
A, eV, (-E_LUMO_)	1.2482
χ, eV, (I + A)/2	3.9389
V, eV, − (I + A)/2	− 3.9389
η, eV, (I-A/2)	2.6907
S, eV, (1/2η)	0.1858
ω, eV, (V^2^/2η)	2.8830
ΔN_max_, (− V/η)	1.4639

**Table 8:** Calculated quantum chemical parameters of the investigated Zn, Cu, and Ni complexes at the B3LYP/GEN level

Compounds	Ni-L	Cu-L	Zn-L
E_T_ (au)	− 2030.1444	− 2210.4148	− 2383.8308
E_HOMO_ (eV)	− 11.7351	− 12.1634	− 12.1381
E_LUMO_ (eV)	− 9.1156	− 7.2979	− 11.6927
E_gap_ (eV)	2.6196	4.8657	0.4454
µ (Debye)	6.7636	0.2258	3.2204
I (eV)	6.4573	12.1641	12.1388
A(eV)	9.1161	7.2984	11.6934
χ(eV)	10.4260	9.7313	11.9161
V(eV)	− 10.4260	− 9.7313	− 11.9161
η(eV)	1.3098	2.4328	0.2227
S(eV)	0.3817	0.2055	2.2449
ω (eV)	41.4943	19.4622	318.7610
ΔN_max_	7.9598	3.9999	53.5009

The very low chemical hardness (η) values of the complexes under investigation suggest that they are capable of efficient charge transport. Substances with lower global nucleophilicity index values and higher global electrophilicity index values are more reactive, in the following order: Zn-L > Ni-L > Cu-L.

### Non-linear optical properties (NLO)

Understanding the mechanisms behind structure–property relationships and determining how to evaluate polarizability and hyperpolarizability are major current pursuits in quantum chemistry. This field heavily relies on the development of novel nonlinear optical materials [65, 66].

The average predicted hyperpolarizabilities and polarizabilities of the compounds under study, as well as the α, ∆α, (µ), and (β) of the synthesized ligand and complexes employing B3LYP/6-311G** and B3LYP/LANL2DZ, are summarized in Table 9.

**Table 9:** Polarizability ( < α˃) and hyperpolarizability ( < β˃) off the ligand and its complexes at the B3LYP/GEN level

Property	Urea	L		
μ, Debye^a^	1.3197	4.0511		
** < **α** > ** × 10^−23^ esu		zero		
Δα × 10^−24^ esu		2.5790		
< β˃ × 10^−30^ esu^b^	0.1947	6.2510		
Complexes		Ni-L	Cu-L	Zn-L
μ, Debye^a^		1.4074	0.2258	3.2204
** < **α** > ** × 10^−23^ esu		zero	zero	− 1.7166
Δα × 10^−24^ esu		1.4162	1.1629	1.5279
< β˃ × 10^−30^ esu^b^		8.3367	7.1239	1.2892

Urea is a commonly used prototype in nonlinear optical (NLO) studies. In this investigation, urea was used as the reference material due to the lack of experimental NLO data for the compounds under consideration. According to the β parameter, the values for the Ni-L, Cu-L, and Zn-L complexes are approximately 42, 36, and 7 times greater than that of urea, respectively, while the investigated ligand exhibits a value about 32 times higher than urea. Therefore, the synthesized ligand and its metal complexes are promising candidates for NLO materials [48, 67–70].

### Molecular electrostatic potentials (MEPs)

The electrophilic and nucleophilic reactivities of the investigated compounds are associated with the negative and positive regions, respectively, which are represented by the colors red and blue [58, 63, 71].

In the complexes, the nitrogen and oxygen groups of the ligand exhibit the most negative (red) regions, while the hydrogen atoms show the most positive (blue) areas. Interestingly, some carbon atoms also display notable electrostatic properties. Regions of the molecule with a negative electrostatic potential are more susceptible to electrophilic attack; the more negative the potential, the higher the likelihood of such an attack occurring. The 3D molecular electrostatic potential (MEP) maps of the ligand and its complexes are presented in Figs. 10 and 11.

### TD-DFT studies

While the Polarizable Continuum Model (PCM) was employed in the TD-DFT theoretical study, the TD-DFT/PCM method was specifically used to simulate the electronic spectra at the same level of theory. PCM treats the solute as being enclosed within a cavity, in contrast to the solvent (dimethylformamide, DMF), which lacks a defined molecular structure. The polarity of the solvent influences the HOMO–LUMO band gap by stabilizing the π* energy levels and destabilizing the π energy levels of the complex. The small band gap observed in the prepared complexes is attributed to the highly polar nature of the DMF solvent used in the calculations [72, 73].

In the PCM method, the solvent is also characterized by its dielectric constant and other macroscopic properties [59, 69, 74]. In the case of a molecule with both occupied and unoccupied molecular orbitals located on the same moiety, the transition should be categorized as internal [75, 76].

**Fig. 12:**
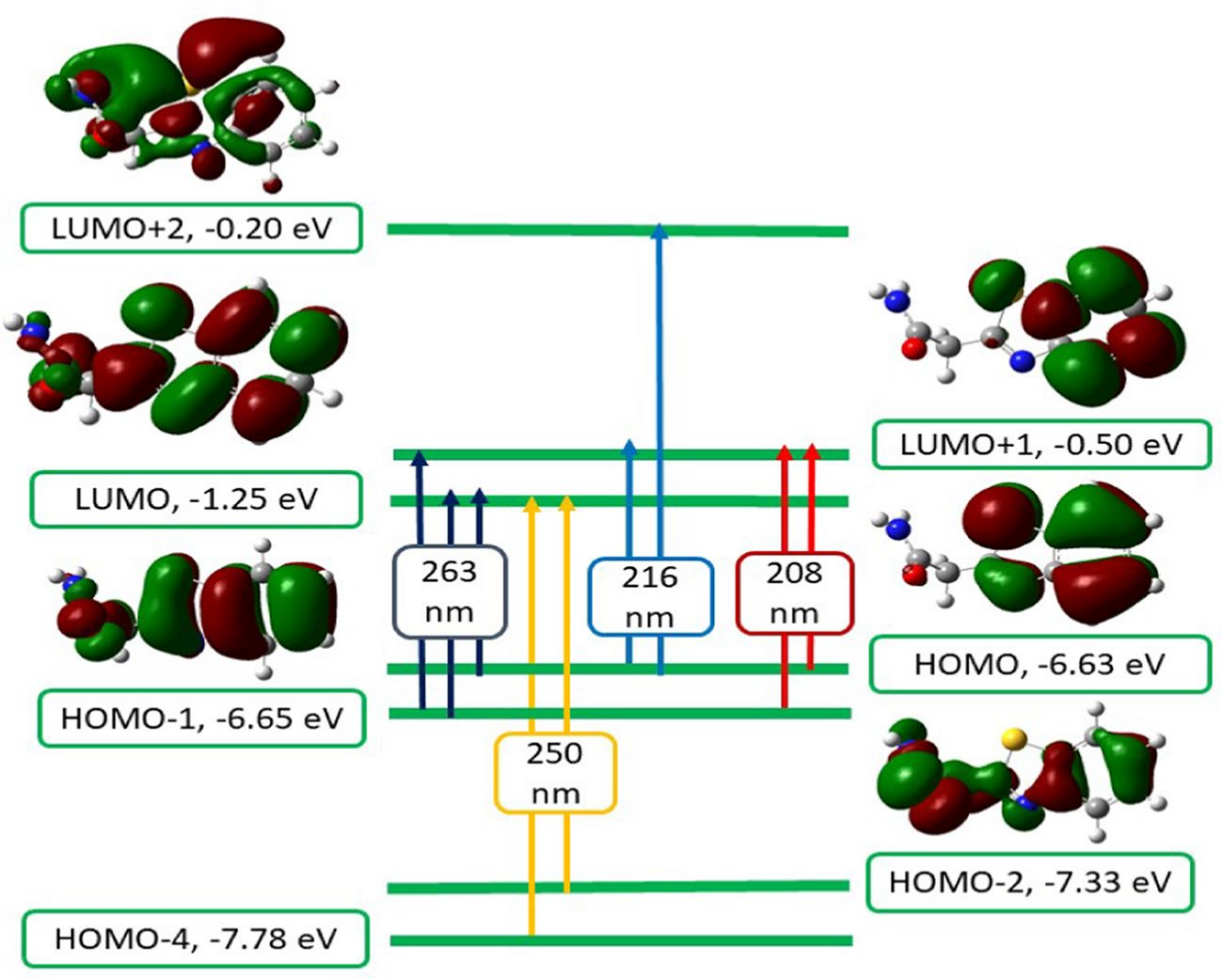
Frontier molecular orbitals and their electronic absorption transitions for the ligand

**Fig. 13:**
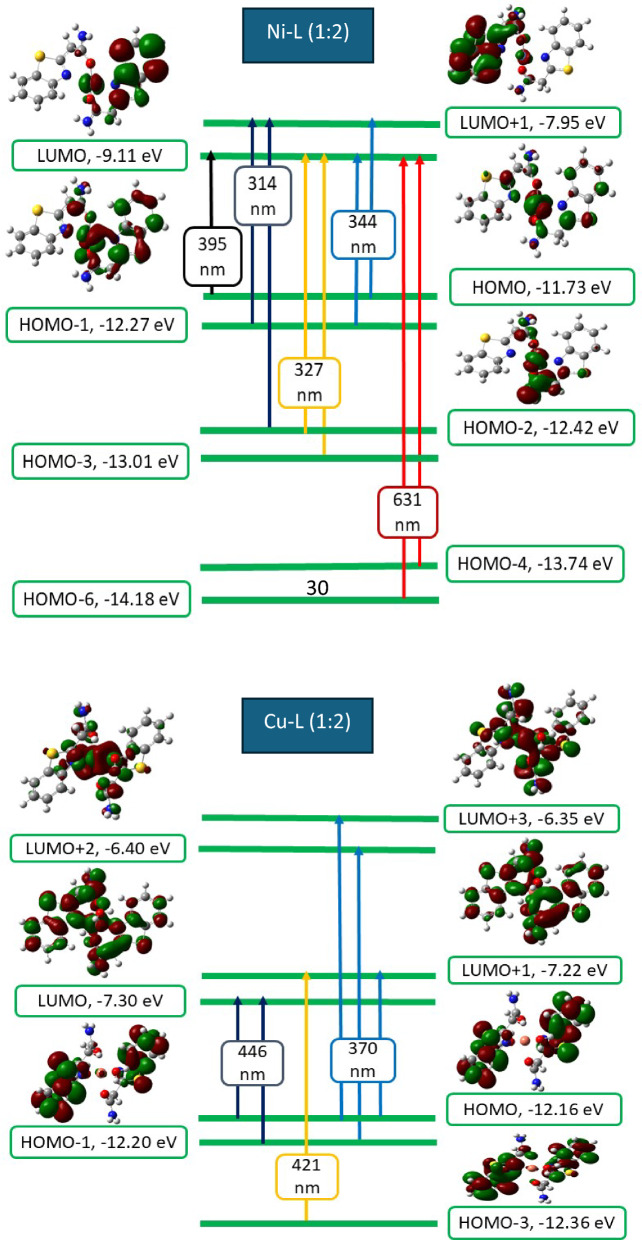
Frontier molecular orbitals and their electronic absorption transitions for Ni and Cu complexes

Figures 12 and 13 show the electronic absorption transitions of the frontier molecular orbitals of the ligand and its complexes, calculated at the TD-B3LYP/6-31G (d,p) level of theory [45, 77–79].

### Molecular docking studies

Molecular docking plays a crucial role in the development of computer-aided modern drugs. MOE2019 software was used to identify the most probable conformations and interaction mechanisms for the NI63 human coronavirus, 1HK7 (breast cancer protein), 6WTT (SARS-CoV-2), and the most active site of 1ACL. Small anti-coronavirus drugs target binding sites on the N protein, making it an attractive pharmaceutical target because these drugs interfere with the protein’s natural oligomerization or RNA-binding functions. This interference helps prevent viral infection [76].

The default configurations and lowest-energy conformers of the docked complexes were used for the docking runs. Molecular docking aims to replicate the process of molecular recognition by minimizing the system's free energy to achieve the optimal conformation of both the protein and the drug (complex) [80].

**Fig. 14.**
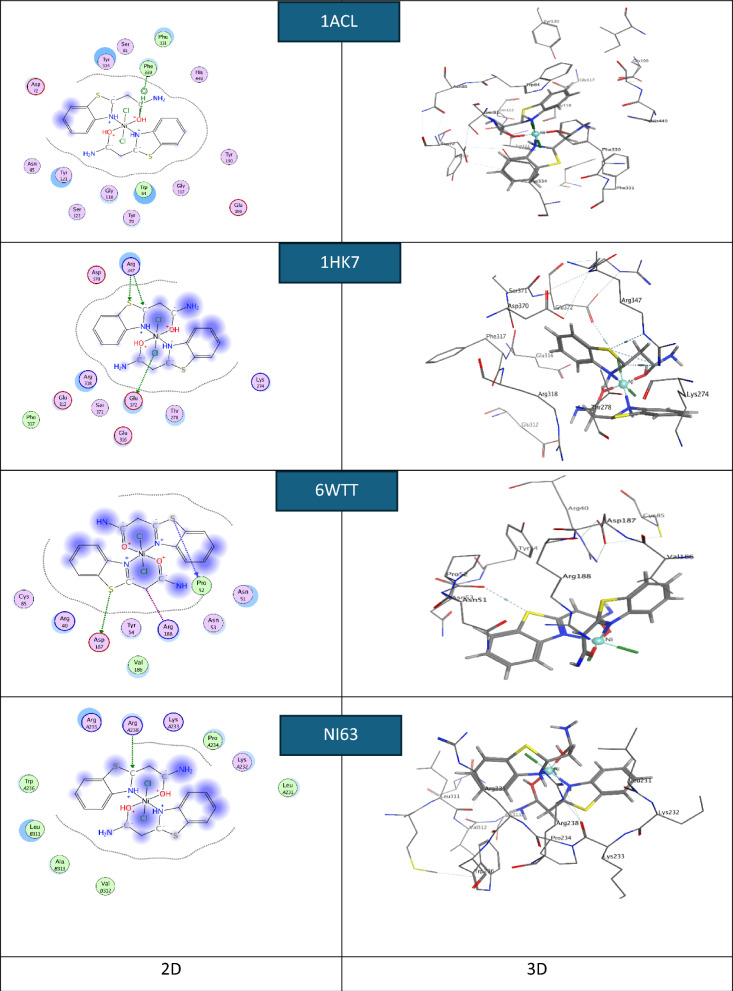
2D and 3D plots showing the interaction between the Ni-L complex and the active sites of the COVID-19 receptors NI63, 6WTT, 1ACL, and 1HK7

**Fig. 15.**
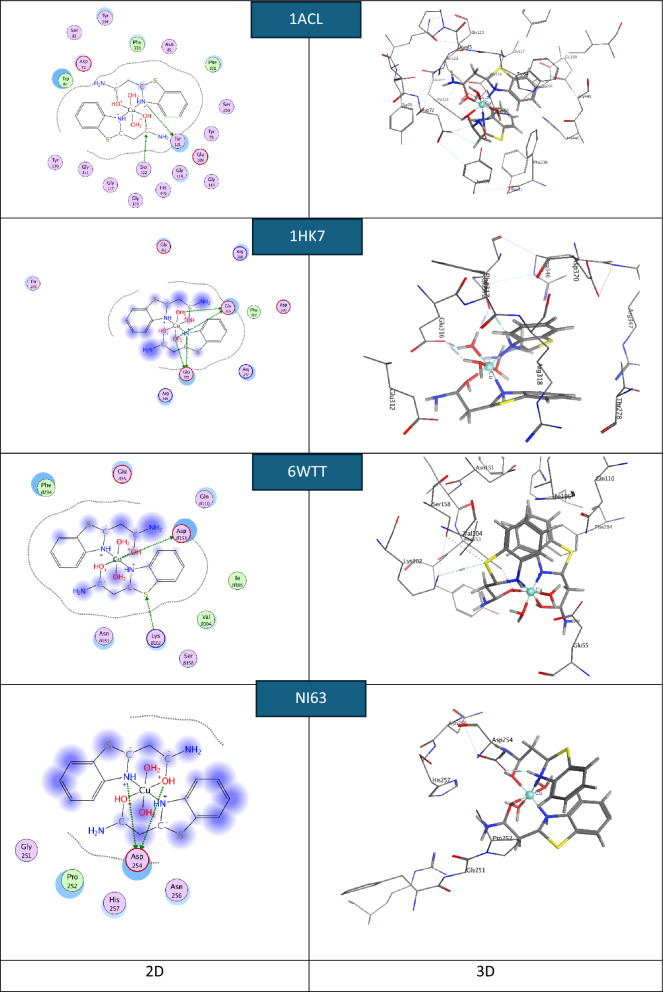
2D and 3D plots showing the interaction between the Cu-L complex and the active sites of the COVID-19 receptors NI63, 6WTT, 1ACL, and 1HK7

**Fig. 16.**
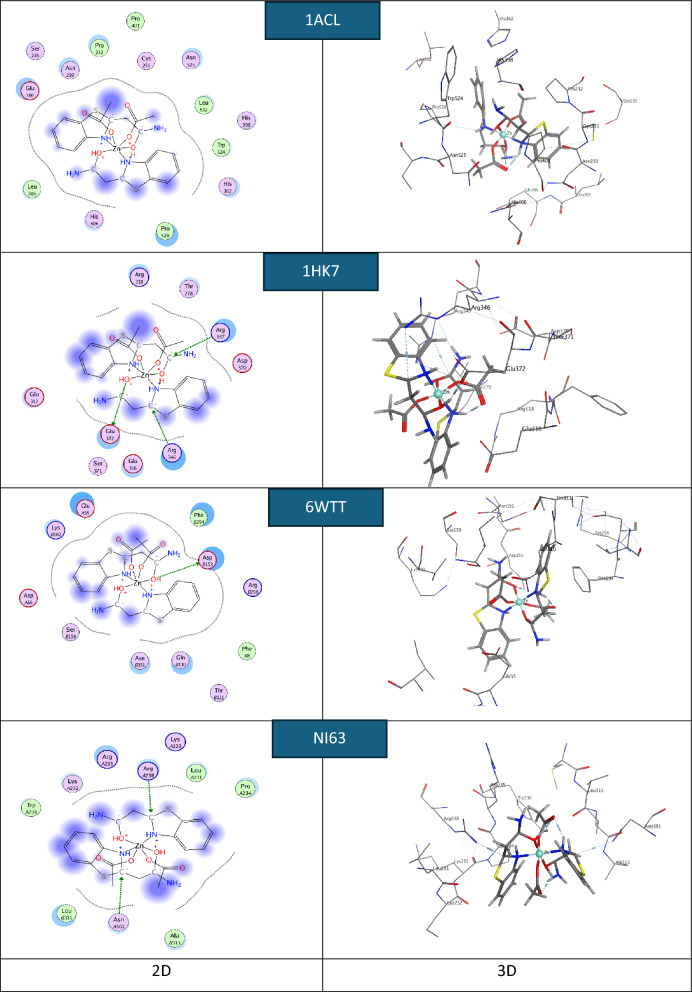
2D and 3D plots showing the interaction between the Zn-L complex and the active sites of the COVID-19 receptors NI63, 6WTT, 1ACL, and 1HK7

In this work, the actual docking phase is simulated using the pairwise interaction energies of the protein complexes. Rotatable bonds are considered to include non-polar hydrogen atoms. Figures 14, 15, and 16 show 3D interaction images of the complexes containing NI63 (human coronavirus) and 1HK7 (breast cancer protein) [81], 6WTT (SARS-CoV-2) [82], and 1ACL (Alzheimer’s) [83–87]. In Figs. 14, 15, and 16, hydrogen bonds are represented by dashed lines. To replicate the actual docking process, Table 10 presents the interaction energies between the complexes and the proteins. Most of the binding interfaces between the complexes and the studied proteins involve ionic interactions, hydrogen bond donors, and hydrogen bond acceptors. Notably, negative binding affinities indicate a strong likelihood of these interactions occurring [85, 88, 89].

**Table 10:** The theoretical docking interaction data between the complexes with the receptors of 1ACL, 1HK7, 6WTT, and COVID-19 NI63

System		Binding score (kcal/mol)	Receptor	Interaction	Distance(Å)	E (kcal/mol)
	Ni-L-NI63	-5.03	ARG 238 (A)	H-acceptor	3.27	− 7.4
	Cu-L-NI63	− 4.75	ASN 301 (A)	H-acceptor	3.50	− 2.3
	Zn-L-NI63	− 4.26	ASP 254 (B)	H-donor	2.71	− 16.0
			ASP 254 (B)	H-donor	2.99	− 10.4
	Ni-L-6WTT	− 5.55	THR 103 (A)	H-donor	3.22	− 2.7
			LYS 102 (A)	H-acceptor	3.54	− 1.3
	Cu-L-6WTT	− 5.06	ASP 153 (B)	H-donor	2.87	− 0.7
			LYS 102 (B)	H-acceptor	3.47	− 3.2
	Zn-L-6WTT	− 6.54	ASP 153 (B)	H-donor	3.20	− 8.6
	Ni-L-1HK7	− 4.80	GLU 372 (A)	H-donor	3.84	− 0.9
			THR 278 (A)	H-donor	3.89	− 0.5
			ARG 347 (A)	H-acceptor	3.92	− 0.7
			ARG 347 (A)	ionic	3.92	− 0.7
			ARG 318 (A)	ionic	3.64	− 1.4
			ARG 318 (A)	ionic	3.56	− 1.7
			ARG 318 (A)	ionic	3.15	− 3.5
	Cu-L-1HK7	− 5.07	GLU 312 (A)	H-donor	3.29	0.2
			GLU 316 (A)	H-donor	2.61	− 26.8
			GLU 372 (A)	H-donor	2.72	− 21.2
			GLU 372 (A)	Ionic	3.52	− 1.8
			GLU 316 (A)	Ionic	3.59	− 1.6
			GLU 316 (A)	Ionic	3.67	− 1.3
			GLU 316 (A)	Ionic	2.61	− 7.7
			GLU 372 (A)	Ionic	2.72	− 6.7
	Zn-L- 1HK7	− 4.90	ARG 318 (A)	Ionic	3.54	− 1.7
			ARG 318 (A)	Ionic	3.61	− 1.5
			ARG 318 (A)	Ionic	3.21	− 3.2
			ARG 347 (A)	Ionic	3.26	− 3.0
	Ni-L- 1ACL	− 6.83	TYR 334 (A)	H-donor	2.95	− 5.4
			TYR 334 (A)	H-donor	3.16	− 6.7
	Cu-L-1ACL	− 4.21	GLU 140 (A)	H-donor	2.77	− 19.4
			GLU 140 (A)	H-donor	2.73	− 28.3
			GLU 140 (A)	Ionic	3.41	− 2.3
			GLU 140 (A)	Ionic	2.93	−5.0
			GLU 140 (A)	Ionic	3.33	− 2.6
			GLU 140 (A)	Ionic	2.73	− 6.6
	Zn-L-1ACL	− 7.33	PRO 232 (A)	H-acceptor	3.60	− 0.7

Due to mutation, the chelates are more likely to bind to the receptor by lowering their binding energy. One of the key features of the complex is the abundance of active hydrogen bonding sites, which enables it to effectively inhibit protein binding [90–92]. This study contributes to the development of more potent inhibitory compounds.

The binding interaction surfaces of the synthesized complexes with the NI63 protein receptor were found to involve hydrogen bond donors and acceptors. These interactions, combined with hydrogen bonding from the docking compounds, contributed to good binding stability. The complexes were observed to bind to the amino acid residues ARG238, ASN301, and ASP254, with the highest docking score of − 5.03 kcal/mol recorded for the Ni complex.

For the 6WTT protein, the interactions also involved hydrogen bond donors and acceptors, with the highest docking score of − 6.54 kcal/mol observed for the Zn complex, which bound to the residues THR103, LYS102, and ASP153.

In the case of the 1HK7 target protein, the complexes interacted with amino acid residues GLU372, THR278, ARG347, ARG318, GLU312, and GLU316 through hydrogen bond donors and ionic interactions. The Cu complex achieved the highest docking score of − 5.07 kcal/mol.

The highest docking score for the 1ACL target protein was − 7.33 kcal/mol, also observed for the Zn complex, which bound through hydrogen bond donor/acceptor and ionic interactions with residues TYR334, GLU140, and PRO232.

The findings indicate that the complexes effectively inhibit the mutant 1ACL protein, which is associated with Alzheimer’s disease. The binding score order for the synthesized compounds is: Zn-L > Ni-L > Cu-L. Additionally, the docking binding scores of the complexes against the proteins NI63 (human coronavirus), 1HK7 (breast cancer protein), and 6WTT (SARS-CoV-2) follow the orders: Ni-L > Cu-L > Zn-L, Cu-L > Zn-L > Ni-L, and Zn-L > Ni-L > Cu-L, respectively.

**Fig. 17:**
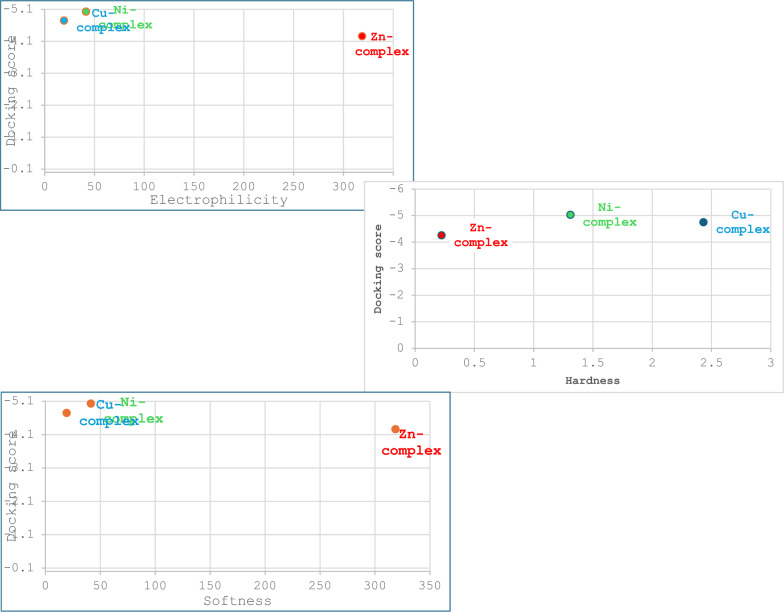
The relationship between molecular docking scores and global reactivity descriptors

Chemical hardness, softness, and electrophilicity are examples of global reactivity descriptors that can be related to molecular docking studies to assess how a molecule’s intrinsic reactivity influences its binding to a receptor. Electrophilicity (ω) reflects a molecule’s tendency to act as an electrophile, attracting electrons and potentially forming bonds with nucleophilic sites in biological proteins. A higher ω value may suggest that a compound is more likely to interact with biological targets. Essentially, a compound with a higher ω value or another relevant reactivity descriptor may be more likely to reach its target, bind efficiently, and produce the desired biological effect. This makes such information highly valuable in drug discovery. Figure 17 shows a plot of global reactivity descriptors against molecular docking scores.

## Conclusion

The study aims to synthesize a benzothiazole derivative and its divalent metal complexes with copper, nickel, and zinc. The structures of the synthesized compounds were confirmed using ^1^H NMR, elemental analysis, ESI–MS, electronic absorption and infrared (IR) spectroscopy, molar conductance, magnetic susceptibility, thermal analysis (TG and DTA), and density functional theory (DFT). The findings can be summarized as follows: According to the IR spectra, coordination occurs through the nitrogen atoms in the heterocyclic ring and the oxygen atoms of the amide carbonyl group, forming bidentate ligands. The molecular structures of the ligand and its complexes were optimized using the B3LYP/6-311G** basis set for non-metal atoms and the LANL2DZ basis set for the metal ions. The compounds exhibit strong nonlinear optical (NLO) characteristics and a small HOMO–LUMO energy gap, making them readily polarizable. Due to their significant polarizability and hyperpolarizability values, the complexes are promising candidates for NLO materials. The TD-DFT/PCM method, employing the B3LYP functional, was used to study electronic transitions via time-dependent density functional theory calculations. Additionally, molecular docking and binding energy studies were conducted with key disease-related proteins, including human coronavirus (NI63), breast cancer protein (1HK7), SARS-CoV-2 (6WTT), and Alzheimer’s disease protein (1ACL). The results demonstrated that the complexes successfully interact with and potentially inhibit these target proteins.

## Supplementary Information

Supplementary material 1.

## Data Availability

All relevant data are included in the manuscript, and supplementary materials are available from the corresponding author upon request.
