# Supplementary material for: Synthesis, characterization, molecular docking studies, and theoretical calculations of novel Ni (II), Cu (II), and Zn (II) complexes based on benzothiazole derivative
Source: BMC Chem. 2025 Jul 4;19(1):202. doi: 10.1186/s13065-025-01576-1 (PMC12228175; doi:10.1186/s13065-025-01576-1)
Supplement: Supplementary file 1 — Supplementary material 1. [file 13065_2025_1576_MOESM1_ESM.docx]

Zn-L

Ni-L

Zn-L

Ni-L

**Figure S1.** The ESI mass spectrum of the Ni and Zn complexes

**
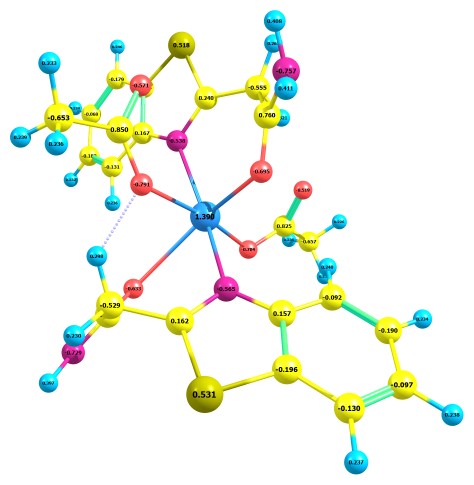

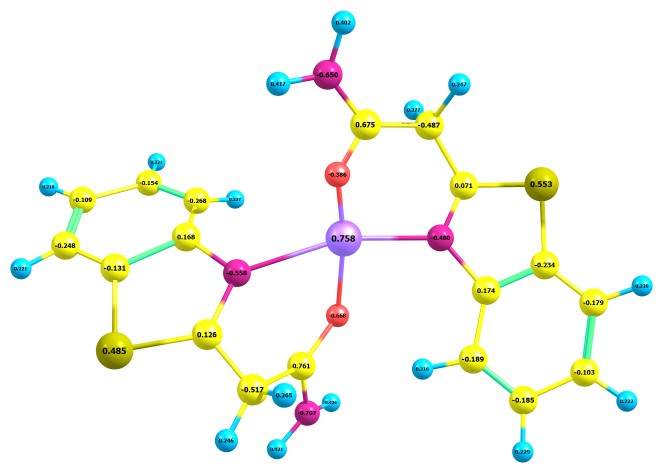

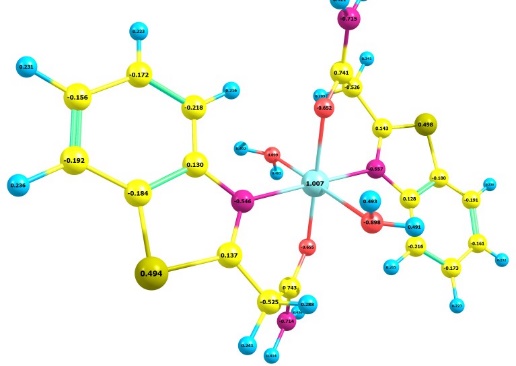
**

Zn-L

Cu-L

Ni-L

**Figure S2.** Natural population charges of the investigated complexes.
